# Combining spinal neuromodulation and activity based neurorehabilitation therapy improves sensorimotor function in cerebral palsy

**DOI:** 10.3389/fresc.2023.1216281

**Published:** 2023-07-26

**Authors:** Rahul Sachdeva, Kristin Girshin, Yousef Shirkhani, Parag Gad, V. Reggie Edgerton

**Affiliations:** ^1^ SpineX Inc., Los Angeles, CA, United States; ^2^ International Collaboration on Repair Discoveries (ICORD), Department of Medicine, University of British Columbia, Vancouver, BC, Canada; ^3^ GirshinPT, Rancho Cucamunga, CA, United States; ^4^ Rancho Research Institute, Downey, CA, United States; ^5^ USC Neurorestoration Center, University of Southern California, Los Angeles, CA, United States; ^6^ Institut Guttmann, Hospital de Neurorehabilitació, Institut Universitari Adscrit a la Universitat Autònoma de Barcelona, Barcelona, Spain

**Keywords:** spinal cord neuromodulation, noninvasive stimulation, cerebral palsy, sensorimotor function, spasticity

## Abstract

Motor dysfunction in individuals with cerebral palsy (CP) such as the inability to initiate voluntary movements, walking with compensatory movement patterns, and debilitating spasticity is due to the aberrant neural connectivity between the brain and spinal cord. We tested the efficacy of noninvasive spinal cord neuromodulation (SCiP™, SpineX Inc.) with activity-based neurorehabilitation therapy (ABNT) in improving the sensorimotor function in six children with CP. Children received 8 weeks of either SCiP™ or sham therapy with ABNT (*n* = 3 per group). At the end of 8 weeks, all participants received 8 weeks of SCiP™ therapy with ABNT. Follow up assessments were done at week 26 (10 weeks after the last therapy session). Sensorimotor function was measured by the Gross Motor Function Measure 88 (GMFM88) test. We observed minimal change in sham group (mean 6% improvement), however, eight weeks of SCiP™ therapy with ABNT resulted in statistically and clinically relevant improvement in GMFM88 scores (mean 23% increase from baseline). We also observed reduced scores on the modified Ashworth scale only with SCiP™ therapy (−11% vs. +5.53% with sham). Similar improvements were observed in sham group but only after the cross over to SCiP™ therapy group at the end of the first eight weeks. Finally, sixteen weeks of SCiP™ therapy with ABNT resulted in further improvement of GMFM88 score. The improvement in GMFM88 scores were maintained at week 26 (10 weeks after the end of therapy), suggesting a sustained effect of SCiP™ therapy.

## Introduction

Cerebral palsy (CP) is the most common childhood motor disorder affecting 2–4 children in every 1,000 births (1–3). The affected children present with a wide range of functional disorders including inability to move voluntarily, maintain balance and posture, spasticity and abnormal sensation during early development that often worsen with age (4, 5). The primary standard of care (SoC) is physical therapy (PT) (6), potentially with subsequent medication and/or surgery to manage pain and reduce spasticity (7). For children with significant spasticity, SoC often includes selective dorsal root rhizotomy (SDR) (8) and intramuscular injections of OnabotulinumtoxinA (9). While these treatments reduce spasticity, they are invasive, may diminish muscle function, and have minimal effect on voluntary sensorimotor function. For instance, 3 months of standard PT resulted in 5.7 points increase in GMFM88 (10); and intramuscular OnabotulinumtoxinA injections result in 1.7–2.2 points increase in GMFM88 after 1–2 months (7). However, CP children that underwent SDR surgery showed a 6.5 points increase in GMFM66 at 4 months (8). More importantly, the GMFM66 decreased by 20 points 17 years post-surgery (11).

Over the last decade, we and others have extensively shown the therapeutic promise of noninvasive spinal cord neuromodulation in spinal cord injury (12–18). We have previously demonstrated the acute (19) and chronic effects (20) of spinal cord neuromodulation on improvements in sensorimotor function in children with CP. However, the effect of activity-based neurorehabilitation therapy (ABNT) alone compared to spinal neuromodulation with ABNT remains unknown. We hypothesized that children with CP who undergo SCiP™ therapy with ABNT will show greater levels of sensorimotor function improvement as assessed by GMFM88 score, compared to children with CP who undergo inactive sham neuromodulation with ABNT. To test this hypothesis, we performed a single blinded, sham-controlled, one-sided crossover study to investigate the impact of noninvasive spinal neuromodulation with ABNT to improve sensorimotor function in children with CP.

## Methods

Six participants diagnosed with CP (GMFCS level I (*n* = 1), level II (*n* = 1), level III (*n* = 1) and level V (*n* = 3), aged 20 months–8 years) were enrolled in the study (Table 1). The participants demographics and baseline characteristics are described in Table 1. Participants were randomly assigned to either treatment or sham group (*n* = 3 each). Sham group received 8 weeks of ABNT with sham therapy (2 mA for 1 min followed by 0 mA for 60 min) whereas the treatment group received 8 weeks of ABNT with therapeutic SCiP™ therapy delivered using our proprietary SCiP™ device (SpineX Inc., Los Angeles, CA) (20). The spinal neuromodulation consists of delayed biphasic waveform formed with a carrier pulse (10 KHz) with a 1 µs delay between the two phases (positive and negative). The delayed biphasic carrier (10 KHz) was combined with a low frequency (30 Hz) burst with a pulse width of 1 ms. Neuromodulation was applied using two adhesive electrodes placed between C5–6 and T11–12 vertebral levels serving as the cathodes (1.25″ in diameter), and two adhesive electrodes over bilateral iliac crests as anodes (3 × 5″). A visible motor contraction of any muscle or any involuntary movement induced by the stimulation, identified by the therapist was used to determine thresholds for the two sites (C5–6: 18–22 mA, and T11–12: 16–20 mA). The neuromodulation intensity was initially set at 20% below the threshold for each site. The intensities over the C5–6 spine ranged between 12 and 18 mA and over the T11–12 ranged between 10 and 16 mA depending on the activity being performed by the participant. During activities involving sitting, rolling, etc., the therapist lowered the amplitudes by 2–4 mA prior to initiation of the activity. Whereas, during standing and stepping, the therapist increased the intensities by 1–2 mA prior to initiation of the activity. During the course of a given activity, the intensities would be modulated ±2 mA based on observed functional performance of the child.

**Table 1:** Demographics, training and descriptive outcomes for the study participants.

	Participant 1	Participant 2	Participant 3	Participant 4	Participant 5	Participant 6
Age	1 year 8 months	2 years 3 months	2 years 11 months	7 years 8 months	3 years 4 months	8 years 2 months
Gender	M	F	F	M	M	F
GMFCS	Level V	Level I	Level V	Level II	Level V	Level III
Group	Treatment	Sham	Sham	Treatment	Sham	Treatment
ABNT activities	BWSTT. Sitting. Floor play. Quadruped and kneeling. Standing.	BWSTT. Standing. Side stepping. Jumping and balance beam.	BWSTT. Sitting. Floor play. Quadruped and kneeling.	BWSTT. Half kneel to standing. Balance beam. Jumping and step ups.	BWSTT. Prone reaching and rolling. Sitting. Quadruped play.	BWSTT. Standing. Sidestepping. Sit to stand. Jumping.
Changes at 8 weeks	Increased head control and accuracy in reaching. Independent rolling and prop sitting.	Sit to stand with no hands.	Increased sitting control and weight bearing on arm in quadruped.	Increased balance in tandem and single leg stances. Ability to jump higher than two inches.	Increased head control and sitting ability.	Independent sit to stand, backward stepping and stair climbing.
Changes at 16 weeks	Independent head control, sitting balance, weight bearing on arms in quadruped, and control in prone.	Increased step length and single leg balance. Symmetrical squat and jump pattern. Independent stair climbing.	Increased sitting balance, floor mobility, and weight-bearing on left arm.	Further increase in balance during tandem and single leg stances. Independent stair climbing.	Increased control in sitting and reaching. Increased forearms control prone and plantar placement in quad & standing.	Walking down stairs with railing support. Maintaining half- kneel position.
Parents’ feedback at the end of 16 weeks	Increased use of the upper extremities. More control in quad position, sitting, reaching, and standing.	Increased balance on uneven terrain and kicking a ball. Increased participation at the playground. Decreased falls.	Increased crawling throughout house. Improved swallowing.	No major carry over effects	Increased ease in sitting postures and increased rolling across the room.	Independent sit to stand and use of stairs. Increased independence in ADLs.

**Figure 1:**
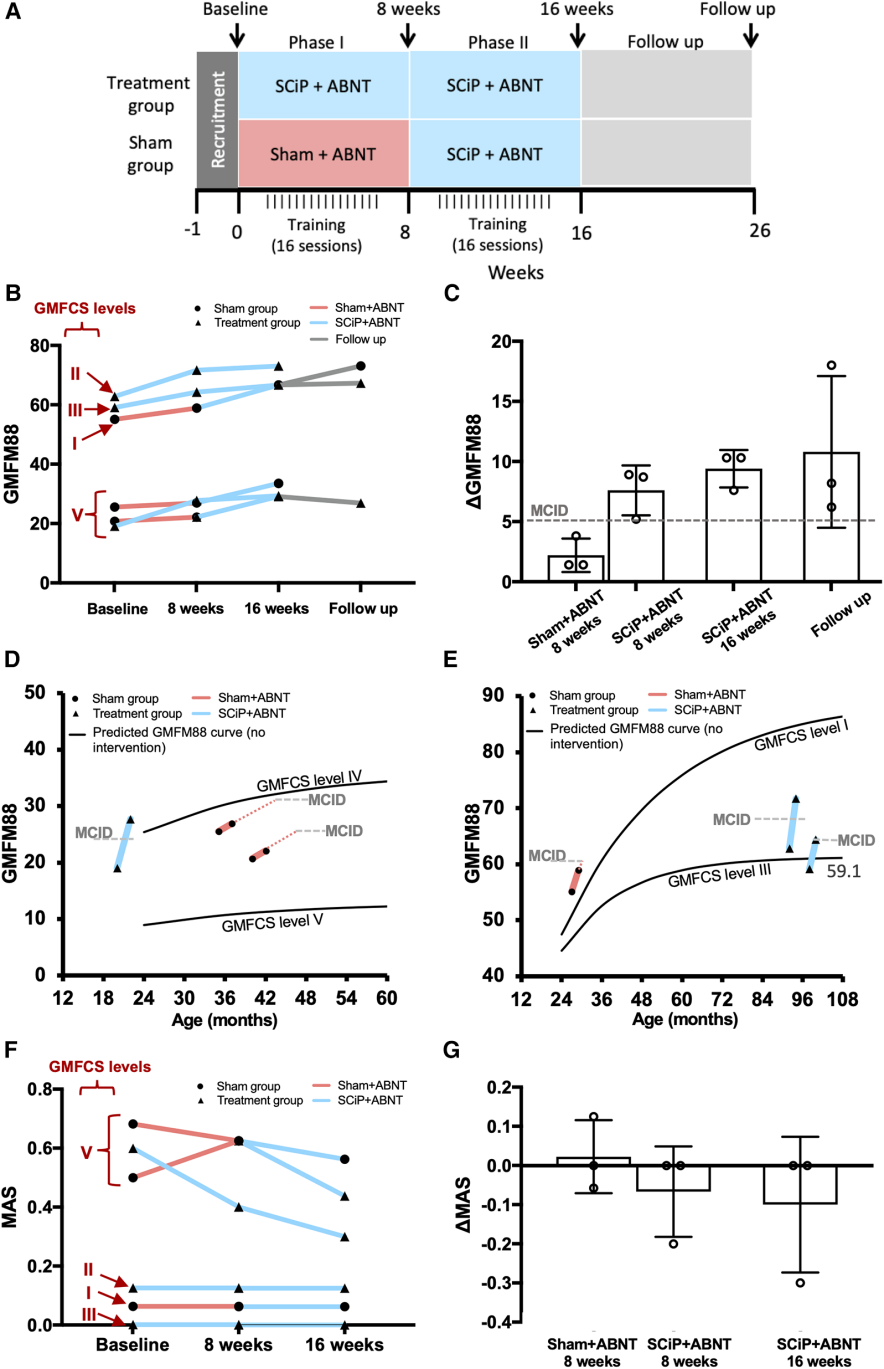
(**A**) Experimental design and timeline of events. (**B**) GMFM88 scores at baseline, week 8, week 16 and follow up. 3 participants started with SCiP™^ ^+ ABNT (blue) vs. 3 with sham + ABNT (red). Sham group crossed over to SCiP™ at week 8 and all participants received SCiPTM from week 8 to 16, followed by 10 weeks of no intervention (grey). (**C**) Mean ± SD change from baseline in GMFM88 scores after 8 weeks of sham, 8 weeks of SCiP™, 16 weeks of SCiP™ and follow up (*n* = 3 each). (**D,E**) Comparison of GMFM88 scores at the end of 8 weeks (primary efficacy endpoint) between sham (red) and SCiP™ (blue) groups with reference to a validated predicted model of change in GMFM scores without an intervention, matched for age and GMFCS level (21). (**F**) spasticity scores (MAS) at baseline, week 8 and week 16 for sham (red) and SCiP™ (blue) groups. (**G**) Mean ± SD change in spasticity scores suggest that children in therapeutic group had lower spasticity at the end of 8 and 16 weeks, compared to baseline.

Details of ABNT sessions are provided in Table 1. The treatment was administered by a trained pediatric physical therapist. The participants (and parents) were blinded to the randomization group. At the end of 8 weeks, the sham group crossed into the therapeutic group and received 8 weeks of SCiP therapy with ABNT. The treatment group continued SCiP™ therapy with ABNT for another 8 weeks (i.e., total 16 weeks; Figure 1A). Voluntary sensorimotor function was measured as the primary outcome using Gross Motor Function Measure 88 (GMFM88) and muscle tone (spasticity) was measured using the Modified Ashworth Scale as the secondary outcome (22), at baseline, 8 weeks, and 16 weeks (10). Ten weeks after the last therapeutic session, three participants were reassessed for the primary outcome. Primary end point assessment was based on improvement in GMFM88 scores at 8 weeks compared to baseline.

## Results

Eight weeks of SCiP™ therapy resulted in an increase (mean ± SD) in GMFM88 scores by 7.6 ± 2.08 points (minimal clinically important difference; MCID = 5 points) (23), compared to a 2.2 ± 1.38 points increase in the sham group (Figures 1B,C). Further, when the participants from sham arm crossed over and received 8 weeks of therapeutic SCiP™, their GMFM88 increased by 7.13 ± 0.6, equivalent to the treatment group. The participants originally randomized to treatment group continued SCiP™ therapy for another 8 weeks and achieved the ΔGMFM88 score of 9.4 ± 1.5 at week 16 compared to baseline. Interestingly, three participants (1 from treatment group and 2 from sham group) that were reassessed at 26 weeks (i.e., 10 weeks after last SCiP™ therapy session and no further intervention) showed a ΔGMFM88 score of 10.8 ± 6.3 compared to baseline, suggesting a sustained effect of SCiP™ therapy with ABNT. All participants receiving SCiP™ therapy qualified as responders at the primary efficacy endpoint (i.e., ΔGMFM88 > 5 points at 8 weeks), and showed an accelerated functional improvement, compared to the predicted GMFM88 model curve matched for age and GMFCS level (Figures 1D,E) (21). Qualitative observations by the physical therapist and parents suggested meaningful functional improvements in response to SCiP™ therapy, during and post treatment. Table 1 describes the qualitative results for each participant, along with notable feedback from parents. Eight weeks of SCiP™ therapy with ABNT reduced spasticity compared to the sham therapy with ABNT group (ΔMAS −0.06 ± 0.1 SCiP™ vs. +0.02 ± 0.09 sham). Continuation of SCiP™ therapy with ABNT for additional 8 weeks further reduced spasticity score (ΔMAS −0.1 ± 0.1). None of the participants demonstrated an increase in spasticity in response to SCiP™ (Figures 1F,G). No adverse events were reported during the course of SCiP™ therapy with ABNT.

## Discussion

To our knowledge SCiP™ therapy with ABNT is the first intervention to show a significant clinical improvement in sensorimotor function in children with CP within a short period of 8 weeks and be able to sustain the improvement for an extended period of time (10 weeks). Our preliminary findings demonstrate greater improvement in sensorimotor function relative to the available standard of care treatment options, reduced spasticity and increased participation in activities of daily living with SCiP™ therapy with ABNT. Although the exact mechanistic understanding of the proposed combination therapy of SCiP™ with ABNT is incomplete, insights can be gained from studies with spinal cord injury and other forms of paralysis. We hypothesize that spinal neuromodulation (SCiP™) transforms the targeted spinal-supraspinal neural networks into an activated state of plasticity, which are made functionally more competent using activity dependent guidance (ABNT), obtained from proprioception (24). The two key findings of this study are (a) the recovery in voluntary motor function even in the absence of active spinal neuromodulation, and (b) the persistence of improved function during the follow up period. While the present study did not directly test the evidence for putative neural plasticity, it has been previously documented in studies investigating neuromodulation-mediated recovery in the spinal cord injury population (25–28). However, since CP and spinal cord injury have distinct pathophysiologies, the mechanism of action responsible for neuromodulation driven changes in sensorimotor function remains unknown. Despite the lack of mechanistic evidence, our initial findings suggest that noninvasive neuromodulation (i.e., SCiP™ therapy) can be a viable option to improve sensorimotor function in CP, and warrants a comprehensive investigation using a randomized control trial with a larger sample size.

## Data Availability

The original contributions presented in the study are included in the article, further inquiries can be directed to the corresponding author.
